# Predictive factors for pregnancy after intrauterine insemination: A prospective study of factors affecting outcome

**DOI:** 10.4103/0974-1208.74154

**Published:** 2010

**Authors:** Mohan S Kamath, Priya Bhave, TK Aleyamma, Raju Nair, A Chandy, Ann M Mangalaraj, K Muthukumar, Korula George

**Affiliations:** Reproductive Medicine Unit, Christian Medical College, Vellore, India; 1 Matha Assisted Reproductive Centre (MARC), Matha Hospital, Kottayam, Kerala, India

**Keywords:** Clinical pregnancy rate, intrauterine insemination, predictive factors

## Abstract

**OBJECTIVE:**

To determine the predictive factors for pregnancy after controlled ovarian hyperstimulation (COH)/intrauterine insemination (IUI).

**DESIGN:**

Prospective observational study.

**SETTING:**

University-level tertiary care center.

**PATIENTS AND METHODS:**

366 patients undergoing 480 stimulated IUI cycles between November 2007 and December 2008.

**INTERVENTIONS:**

Ovarian stimulation with gonadotrophins was initiated and a single IUI was performed 36 h after triggering ovulation.

**MAIN OUTCOME MEASURES:**

The primary outcome measures were clinical pregnancy and live birth rates. Predictive factors evaluated were female age, duration of infertility, indication for IUI, number of preovulatory follicles, luteinizing hormone level on day of trigger and postwash total motile fraction (TMF).

**RESULTS:**

The overall clinical pregnancy rate and live birth rate were 8.75% and 5.83%, respectively. Among the predictive factors evaluated, the duration of infertility (5.36 vs. 6.71 years, *P* = 0.032) and the TMF (between 10 and 20 million, *P* = 0.002) significantly influenced the clinical pregnancy rate.

**CONCLUSION:**

Our results indicate that COH/IUI is not an effective option in couples with infertility due to a male factor. Prolonged duration of infertility is also associated with decreased success, and should be considered when planning treatment.

## INTRODUCTION

A combination of controlled ovarian hyperstimulation (COH) with intrauterine insemination (IUI) remains an important option available to an infertility specialist and is a widely used treatment modality for a broad range of indications. Common indications include cervical factor, mild endometriosis, mild to moderate male factor, ovulatory dysfunction and unexplained infertility.[1] The reported pregnancy rates per cycle range from 8 to 22%.[2–4] The pregnancy rates per IUI cycle are quite variable in the literature due to differences in cause and duration of infertility, concomitant usage or omission of ovarian stimulation, sperm preparation techniques, treatment cycles and number of times IUI is performed during a cycle (single or double).[1–4]

The cumulative pregnancy rates for COH with IUI vary according to the indications, and are in the range of 20–33%.[4,5] Generally, four cycles of COH/IUI are recommended depending on the female age prior to moving on to assisted reproductive technology (ART)-*in vitro* fertilization (IVF).[3,6]

Several prognostic factors with regards to IUI treatment outcome have been identified, and include factors such as patient profile, duration of infertility, type of infertility, stimulation protocol, follicular response, endometrial thickness, timing of IUI and semen parameters, like postwash motility, morphology and total motile fraction (TMF).[4,7–10]

Even though COH/IUI is a less expensive and simpler form of treatment compared with ART-IVF, it still requires frequent monitoring and supervision under specialist care.

The knowledge that the per-cycle pregnancy rates are not very encouraging combined with the time, effort and financial implications of such treatment, attempting a COH/IUI cycle could be a challenging decision for the couple. Many couples would opt for one attempt of IVF rather than undergo three to four cycles of COH/IUI. This is especially true for couples travelling long distances seeking treatment in other cities/centers.

Since ours is a tertiary care center with couples coming from different cities/towns for treatment, we decided to analyze the variables that contribute to the success of stimulated intrauterine cycles. A prospective study was planned to determine prognostic factors for IUI in our setting that would provide important data for predicting the success of therapy and further help in planning subfertility treatment for the couples.

## PATIENTS AND METHODS

This prospective, observational study was conducted at a university-level tertiary care center.

The study group comprised of couples with male factor infertility, unexplained infertility, minimum to mild endometriosis and anovulation. Patency of at least one tube, confirmed by diagnostic laparoscopy or hysterosalphingography (HSG), was obligatory.

Male factor infertility: defined as semen concentration <20 million sperms/ml, normal morphology <30% (WHO criteria) and progressive motility <50% (A + B) before sperm preparation as per the WHO (1992) guidelines.

Unexplained infertility: couples for whom the results of a standard infertility evaluation are normal (standard tests includes a normal semen analysis [WHO 1992], ovulatory cycles and a HSG or laparoscopy showing patent tubes).

Minimal endometriosis: score 1–5; mild endometriosis: score 6–15 by the revised AFS criteria.

Exclusion criteria included those patients with bilateral tubal blockage, moderate to severe endometriosis and severe male factor infertility, with TMF postwash of <1 million.

However, those patients with a lower sperm count after preparation on the day of IUI were included.

The protocol for ovulation induction included either gonadotrophin only (Menogon; Ferring Pharmaceutical, India) or a combination of clomiphene citrate (Fertomid; CIPLA, India) with gonadotrophin (hMG [human menopausal gonadotrophin]). In the clomiphene and hMG group, tablet clomiphene citrate 100 mg was started from day 2 of menstrual cycle for 5 days with inj. hMG 75 IU being administered on alternate days from day 5 (day 5, 7 and 9).

In cycles stimulated with gonadotrophin alone, inj. hMG 75 IU was administered on a daily basis from day 5 to day 9. On day 10 of stimulation, assessment of follicular development was performed using transvaginal ultrasound. Further stimulation with gonadotrophin was determined according to follicular response. The aim of stimulation was to achieve a monofollicular response. Once a follicle of >17 mm size was identified, a serum luteinizing hormone (LH) test was performed: if the LH level was <15 IU/ml, inj. human chorionic gonadotrophin 5000 IU (hCG) was given as an ovulation trigger and a single IUI was planned 36 h later. If the LH level was >15 IU/ml, hCG trigger was not given and an IUI was performed the next day.

If four or more mature follicles (>17mm) developed, the cycle was cancelled and the couples were advised to avoid intercourse for the next 2 weeks.

On the day of IUI, the husband was instructed to give a semen that was prepared by the double-density gradient method with Puresperm (Nidacon, International AB, Gothenburg, Sweden). The postwash sample parameters were assessed. Under aseptic precautions, IUI was carried out with a soft IUI catheter (Sunn Catheter; SAR Healthcare Ltd., India) with an insemination volume of 0.5ml. After the procedure, the patient was advised 20 mins of bed rest. All women were provided luteal phase support with natural micronized progesterone vaginal pessaries for 16 days.

If menstrual cycle was delayed, urine pregnancy test was carried out. When positive, a transvaginal ultrasound was performed 2 weeks later to confirm a clinical pregnancy.

Clinical pregnancy was defined as the presence of an intrauterine gestational sac, confirmed by ultrasound. Higher-order pregnancies were defined as three or more gestational sacs visualized at ultrasound.

Women were followed till delivery and the neonatal outcome was recorded. The primary end point was clinical pregnancy and live birth rate per cycle.

### Statistical analysis

The variables selected were patient parameters like age of woman, duration of infertility, type of infertility and cause of infertility. Parameters related to ovulation induction, like number of dominant follicles, endometrial thickness, LH level on the day of trigger and duration between trigger and IUI were recorded. Laboratory parameters like days of abstinence, post-wash motility, morphology and TMF were recorded.

Of these parameters, age of the woman, duration of infertility and days of stimulation were recorded as continuous variables, and their means were compared using the independent *t*-test.

Other parameters were taken as categorical variables and were compared for significant differences using the Chi-square test.

A *P*-value of <0.05 was considered to be significant.

Data were entered in SPSS version 11 (SPSS Inc., Chicago, IL, USA).

## RESULTS

We evaluated 366 patients undergoing a total of 480 stimulated IUI cycles between November 2007 and December 2008.

Of the 480 homologous COH/IUI cycles analyzed, 346 were the first-treatment cycle, 102 were the second-treatment cycle, 28 were third-treatment cycle and four were fourth-treatment cycle during the study period.

The mean female age was 30.04 ± 4.295 years (range, 20–43 years).

Cause of infertility was anovulatory in 23.8%, male factor in 20.4%, endometriosis in 8.5% and unexplained in 47.3% of the patients.

The categorical variables were compared using the Chi-square test. Of all the categorical variables, the only significant variable affecting outcome was TMF (*P* = 0.002) [Table 1].

**Table 1:** Factors affecting pregnancy rates in intrauterine insemination

Parameters	Pregnancies/cycle	Pregnancy (%)	Chi square
Infertility etiology			0.494
Anovulation	11/103	10.6	
Male factor	5/93	5.37	
Endometriosis	3/38	7.89	
Unexplained	23/202	11.38	
Type of infertility			0.916
Primary	32/358	8.93	
Secondary	10/79	12.6	
Number of preovulatory follicles			0.303
1	29/345	8.52	
2	10/753	13.33	
3	3/14	21.4	
LH level (IU/ml)			0.970
<5	15/124 (12.04)	12.04	
5–9.9	14/133 (10.52)	10.52	
10–14.9	5/48 (10.41)	10.41	
>15	5/76 (6.57)	6.57	
Time between trigger and IUI			0.293
≤24 h	3/73 (4.10)	4.10	
>24 h	38/339 (11.20)	11.20	
Endometrial thickness (mm)			0.787
<6	2/32	6.35	
>6	40/402	9.95	
Days of abstinence			0.885
<2 days	0/12	0	
2–7 days	38/393	9.66	
7–10 days	2/23	8.69	
Motility (%)			
<60	13/208	6.25	0.259
60–80	13/130	10	
>80	14/94	14.89	
Morphology (%)			0.911
<30	1/16	6.25	
>30	39/416	9.37	
Total motile fraction (million/ml)			0.002
<5	3/110	2.7	
5–10	4/71	5.63	
10–20	15/82	18.29	
20–50	12/91	13.18	
>50	7/85	8.23	

The continuous variables were compared using the independent *t*-test. The duration of infertility was found to be significantly associated with the chances of success (5.36 vs. 6.71 years, *P* = 0.032) [Table 2].

**Table 2:** Factors affecting the pregnancy rates in intrauterine insemination

Parameters	Pregnancy		*P* value
	Yes	No	
Age (years)	29.29 ± 3.90	30.31 ± 7.55	0.384
Duration of infertility (years)	5.35 ± 2.77	6.72 ± 3.88	0.032
Days of stimulation	7.19 ± 2.09	6.93 ± 2.76	0.631

The overall pregnancy rate per cycle was 8.75% (42/480). Of these, 41 pregnancies were clinical (8.54%), 7 resulted in abortions (17%) and two were ectopic pregnancies (4.7%). One pregnancy was biochemical in nature. There were no multiple pregnancies.

Of 7 abortions, 6 were first trimester and 1 was second trimester. Of 32 pregnancies that went on to term, 28 patients were followed-up while 4 patients could not be traced. 18 patients underwent Caesarean section and 10 had normal vaginal delivery. A total of 17 boys and 11 girls were delivered. No major congenital anomaly or neonatal mortality was recorded. The live birth rate/cycle was 5.83% (28/480) [Table 3].

**Table 3:** Pregnancy outcome of the intrauterine insemination cycles

Parameters	Outcome/cycle (%)
Pregnancies/cycle	42/480 (8.75)
Live births	28/480 (5.83)
Miscarriages	7/42 (17)
Ectopic pregnancies	2/42 (4.7)
Multiple pregnancies	Nil

A multivariate analysis could not be carried out as the variables found to be significantly affecting outcome were only two.

## DISCUSSION

In our study, we made an effort to determine the prognostic factors that would determine the success of COH/IUI. The variables selected were patient parameters like age of the woman, duration of infertility, type of infertility and cause of infertility. Parameters related to ovulation induction included number of dominant follicles, endometrial thickness, LH level on the day of trigger and time duration between trigger and IUI. Laboratory parameters like days of abstinence, postwash motility, morphology and TMF were recorded.

Among the patient parameters, female age is important as declining oocyte quality associated with increasing age is well documented.[11,12] Even more-effective treatment options like ART-IVF cannot completely overcome the negative impact of age.[13] In our study, a trend toward reduction in success rate with COH/IUI was noted in women with age >35 years, although the difference was not statistically significant. However, many studies have documented a significant drop in the success rate beyond the age of 40 years, with reported live births being as low as 1.4%.[6,14,15] Put together, for women over 35, COH/IUI as a treatment option needs careful consideration, and for women over 40, IUI is a poor treatment option.

The success rate was significantly lower, with an increase in duration of infertility (5.36 vs. 6.71 years, *P* = 0.032). An earlier study also found a significant decline in the success of IUI therapy as the duration of infertility increased.[10] However, we were unable to determine any particular cut-off beyond which IUI could be discouraged. With increasing duration of infertility, IUI as an option appears to be less-effective.

No difference was noted in the success rate with regards to the type of infertility. Among indications for IUI, the success rate was higher in anovulatory and unexplained infertility patients as compared with endometriosis and male factor infertility, although the difference did not reach statistical significance. The trend toward lower pregnancy rates in endometriosis has been documented in an earlier meta-analysis, with the pregnancy rates reduced to half in comparison with other infertility indications.[7] The pregnancy rate in our study for male factor infertility was marginally lower than that in previously reported studies.[3,9] Our overall pregnancy rate with COH/IUI of 8.75% per cycle is low as compared with the results from other studies.[3,9] Our policy of performing only homologous IUI could be one of the reasons for lower success rates as pregnancy rates of up to 22% per cycle are documented when donor insemination is carried out.[3,10]

Another important reason for lower pregnancy rates in our setting could be our strategy of aiming for monofollicular development during COH. This, while leading to lower pregnancy rates, reduces both the multiple pregnancy and the hyperstimulation rates. With the current trend of limiting the number of embryos transferred in IVF cycles, ovulation induction is emerging as the prime cause for higher-order pregnancies. Earlier studies have reported an incidence of 20% twins and 39% higher-order multiple pregnancies as a result of ovulation induction, outside ART.[16,17] All the recorded clinical pregnancies following COH/IUI in our center were singleton, and no case of hyperstimulation was documented during the study period. In our study, a monofollicular response was observed in 71.87% of the cycles. With an increase in the number of preovulatory follicles, a corresponding increase was observed in the pregnancy rates, as shown in Table 1. Studies have shown that the cycle fecundity rates are higher (20–33%) when superovulation protocols are used.[6,8] However, multiple pregnancy rates, including higher-order pregnancies, also increase with superovulation protocols. Finally, each unit/center needs to decide regarding the stimulation protocols/policies and try to achieve a balance between the quest for a higher success rate and acceptable multiple pregnancy rates.

We found a trend toward a higher pregnancy rate with endometrial thickness >6 mm; however, the difference was not statistically significant.

The incidence of premature LH surge in COH/IUI is in the range of 24–36%, and negatively influences the outcome of the treatment cycle.[18] In our study, the incidence of premature LH surge (>15 miu/ml) was 15.83% (78/480). The pregnancy rates were lower in patients with premature LH surge (6.57%), which is in agreement with earlier studies.[19] Studies looking at the role of antagonist in the prevention of an LH surge in COH/IUI cycles have reported pregnancy rates of up to 20–35%, although the rise in the cost factor needs to be carefully considered.[18,20] The role of clomiphene citrate as a cost-effective alternative in the prevention of an LH surge has been looked at in an earlier study, and promising results have been obtained.[21]

The duration of period of abstinence did not influence the pregnancy rates. Even though no pregnancies were recorded in the group with an abstinence period of <2 days, it is difficult to draw conclusions since the numbers in that group were small (*n* = 12). Even though studies have shown an increase in the ejaculated volume, sperm count and motility with longer periods of abstinence, this has not been shown to improve the IUI pregnancy rates.[22]

The TMF is an important prognostic factor for IUI success. We found a significantly higher pregnancy rate (18.29%) when the TMF was in the range of 10–20 million. The pregnancy rate was lower when the TMF was in the range of 5–10 million (5.63%), with disappointing rates (2.7%) when the TMF was <5 million. Unexpectedly, the pregnancy rates were also lower with high TMF (>20 million). In one of the earlier studies, the authors tried to arrive at a cut-off with regards to seminal parameters at which IUI would be of benefit in male factor infertility.[9] A TMF of <1 million was associated with poor pregnancy rates. When the TMF was <5 million, sperm morphology appeared to play an important role. A pregnancy rate of 5.43% was observed with morphology of <30% as compared with 18.42% with a normal morphology.

Combining the finding of our study and previous reports, we believe that in male factor infertility with a TMF <5 million, couples should be carefully counselled and the option of ART-IVF should be offered more liberally, especially if the female partner’s age is advanced.

We obtained a pregnancy rate/cycle of 8.75% (42/480). The live birth rate/cycle was 5.83%, which is lower than that reported previously.[23,24] No major congenital anomaly was recorded, nor was there any multiple pregnancy.

The purpose of our study was to determine prognostic factors for predicting IUI success. We found only two parameters significantly affecting success – duration of infertility and TMF. In an earlier study, the authors found five prognostic factors, namely number of treatment cycles, number of preovulatory follicles, age, etiology and duration of infertility.[3] A larger study on similar lines would help identify more predictors and also develop a prediction model for pregnancy following IUI cycles.[25] Once a prediction model is developed, validation studies could be carried out to prove its robustness. This could help formulate guidelines and make it easier for couples and clinicians to take important decisions regarding the all-important next step during the course of infertility treatment.

## CONCLUSION

COH/IUI is an important treatment option for varied indications, especially when female age is <35 years. Definitive prognostic factors for predicting success will help in counselling patients regarding the modality of treatment. Significantly higher pregnancy rates were observed when the duration of infertility was <5 years, and the TMF was between 10 and 20 million. Low pregnancy rates were associated with poor semen parameters, indicating that COH/IUI is not an effective option in these clinical situations. The overall pregnancy rate per cycle with COH/IUI in our study was 8.75% and live birth rate per cycle was 5.83%. Our policy of conducting only homologous IUI cycles together with a monofollicular ovulation induction approach are possibly responsible for the comparatively low pregnancy rates obtained. Avoiding twin and higher order pregnancies would be the advantage of such an approach. Many of the identified variables were not shown to significantly affect the outcome. Perhaps, a larger sample size may help in formulating a better predictive model for IUI success. The information could be used by couples and clinicians during counselling to arrive at a decision with regards to their treatment options.
